# Mapping the Conformational Landscape of DNA Minicircles Through Atomic Force Microscopy and Shape Space Analysis

**DOI:** 10.1002/smll.202514267

**Published:** 2026-05-14

**Authors:** Laura Wiggins, Tobias A. Firth, Max C. Gamill, Thomas E. Catley, Agnes Noy, Jonathan M. Fogg, Lynn Zechiedrich, Alice L. B. Pyne

**Affiliations:** ^1^ School of Chemical Materials and Biological Engineering University of Sheffield Sheffield UK; ^2^ School of Physics Engineering and Technology University of York York UK; ^3^ Departments of Molecular Virology and Microbiology Baylor College of Medicine and Biochemistry and Molecular Pharmacology Houston Texas USA

**Keywords:** AFM Image analysis, DNA conformation, machine learning, MD simulations

## Abstract

The structural dynamics of DNA underpin essential biological processes, yet conventional structural biology methods often obscure conformational heterogeneity through ensemble averaging. Atomic force microscopy (AFM) provides single‐molecule topographical maps capable of capturing both local and global variation, but extracting quantitative insight from these images remains challenging. Here, we introduce an automated framework that reduces AFM data to spline representations of the DNA backbone and applies cyclic Procrustes analysis to quantify shape similarity across ensembles. Using purified topoisomers of 339 bp DNA minicircles ranging from relaxed to highly negatively supercoiled, we resolved and measured the relative abundance of conformational states across the different topoisomers, capturing gradual transitions among open circles, compact conformations, and self‐crossing structures that are invisible to techniques such as gel electrophoresis or cryoelectron microscopy (cryo‐EM). We show that beyond quantification, Procrustes distances provide supervisory signals for neural network training, enabling feature extraction tuned to conformational geometry and supporting robust conformation classification of AFM images. Extending the same spline representation to molecular dynamics simulations allows experimental and computational ensembles to be directly compared, establishing a common shape‐based framework for probing conformational variability. Together, these advances transform AFM from a descriptive imaging tool into a quantitative platform for mapping conformational continua, with broad applicability to DNA and other dynamic biomolecular systems.

## Graphical abstract

An automated framework is presented for quantifying DNA conformational variability from atomic force microscopy (AFM) images. DNA backbones are reduced to spline representations and analyzed using cyclic Procrustes analysis to measure shape similarity across ensembles. The approach resolves conformational states of DNA minicircles, guides neural network image classification, and enables direct comparison between experimental AFM data and molecular dynamics simulations.

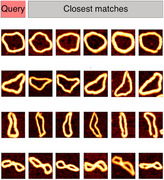

## 1 Introduction

Recent advances in techniques such as X‐ray crystallography [1], NMR [2], cryo‐EM [3], and cryo‐ET [4] have dramatically transformed our understanding of macromolecular structures, including DNA conformations. These approaches, however, fail to capture the dynamic conformations and structural heterogeneity of single molecules of DNA [5]. This limitation arises from their reliance on ensemble averaging, through crystallization, the superposition of NMR spectra, the alignment of cryo‐EM images, and the methods rendering the samples static for X‐ray crystallography, cryo‐EM, and cryo‐ET, which inevitably masks the structural variability present at the single‐molecule level [6]. Atomic force microscopy (AFM) offers unique advantages for studying DNA dynamics, providing high signal‐to‐noise topographical maps of single‐molecules under near‐native physiological conditions [7, 8]. Unlike other techniques, AFM does not rely on ensemble averaging or constraining molecules, allowing structural flexibility so that DNA bending, looping, and supercoiling can be directly visualized. Whereas cryo‐ET can provide 3D information on individual minicircles the resulting structures are of low resolution. Without ensemble averaging, there is a limit on the resolution obtainable with cryo‐ET. In comparison, advances in AFM continue to improve resolution of individual minicircles [9, 10]. However, the capacity of AFM to visualize both local and global heterogeneity in DNA geometry introduces the challenge of developing analytical approaches to quantify these differences.

One such approach for single‐molecule quantification is classical feature extraction, as facilitated by TopoStats, which extracts information on molecular shape, size, and topographical features such as height and volume, enabling comparison across different sample types [11, 12]. The use of such features are advantageous in being readily human‐interpretable, but they may fail to capture subtle conformational differences between molecules and typically require prior assumptions about which descriptors will be informative for a given analysis [13]. Moreover, additional analytical approaches are often required to combine these features effectively in order to identify meaningful groupings and uncover structural heterogeneity within a dataset.

DNA as found in cells is typically maintained in a looped and negatively supercoiled (underwound) state, which has a profound effect on its properties and activities. The torsional strain associated with negative supercoiling leads to disruptions in base pairing and these can range from a single base flipped out of the helix to more extensive denaturation including stretches of single‐stranded DNA [14]. These distortions generate hyperflexible hinges that can facilitate sharp bending. Combined with the free energy provided by supercoiling, base‐pair disruptions allow the DNA to adopt a wide distribution of 3D conformations, reproducible depending upon the degree of under‐ or over‐winding, that would be energetically unfavorable in relaxed DNA [15]. Base pair disruptions are often found at the apices of supercoiled DNA where the DNA is most sharply bent [14, 16]. Conversely, the bending strain associated with sharp bending also directly disrupts base pairing [14]. Supercoiling can also transmit mechanical stress along the DNA backbone to disrupt base pairing at distant sites [14]. The dynamics of supercoiling‐ and looping‐promoted DNA conformations has remained elusive. The development of high‐resolution techniques to observe DNA on a single‐molecule level is beginning to reveal the remarkable structural diversity of supercoiled DNA.

The elastic free energy provided by DNA supercoiling may produce a continuum of conformations, discrete states, or a mixture of both. Characterizing supercoiled DNA minicircle dynamics, therefore, necessitates analytical strategies that move beyond categorical classification and instead quantify the degree of similarity and variability across conformational ensembles. This challenge mirrors those faced in computer vision, where recognizing faces or handwritten characters requires sensitivity to subtle shape differences within the same class. In both cases, progress depends on methods that move beyond assigning labels to quantifying the continuous variation in shape. Here, we apply this principle to AFM images of DNA molecules, adapting shape analysis and pattern recognition strategies to probe conformational heterogeneity. We used 339 bp minicircles [14, 16, 17] for this study for several reasons. Short supercoiled DNA loops are frequently found in nature and 339 bp minicircles are representative of these loops. The small size of the minicircles allows for isolation of pure preparations of minicircles with a given linking number (*Lk*), which is defined by the number of times two strands coil about one another. A detailed description of *Lk* and superhelical density can be found elsewhere [14, 17]. Despite their small size, we are able to generate and purify six different negatively supercoiled topoisomers (Δ*Lk* = ‐1 to ‐6), in addition to the relaxed topoisomer (Δ*Lk* = 0), to explore, categorize, and quantify the dynamics of the wide variety of shapes of negatively supercoiled DNA.

Achieving this type of shape comparison requires an image processing pipeline that extracts molecular contours from raw AFM images, providing a representation of the DNA backbone suitable for quantitative analysis. Our previous work has established a robust pipeline for tracing DNA molecules in AFM images, capable of handling even complex topological species with high accuracy [12]. The pipeline generates spline representations of single molecules that capture both local and global structural detail, preserving this information for meaningful conformational comparison. In this work, we present a pipeline that uses the molecular splines derived from AFM images as input for cyclic Procrustes analysis [18]. This method normalizes and aligns splines to eliminate differences in scale, orientation and contour starting point, and subsequently computes the Procrustes distance that quantifies geometrical similarity between molecules, with smaller Procrustes distances indicating greater shape similarity and larger values reflecting greater dissimilarity [19] Procrustes analysis has already proven a powerful technique in biological contexts ranging from cell morphology to tumor classification [20, 21]. Yet, to our knowledge, this represents the first application to AFM images of DNA, owing to the unique challenges of reducing raw AFM images to spline representations suitable for input.

To validate our approach, we assess the conformational heterogeneity induced by DNA supercoiling, a process that drives changes ranging from major alterations in global conformation to subtle local deviations, all of which profoundly influence fundamental biological processes including transcription, replication, chromatin organization, recombination, and DNA repair [22]. Previous studies have demonstrated that increased supercoiling of minicircles gives rise to distinct conformational classes, characterized by increased compaction and self‐crossing relative to the open minicircles typical of the relaxed ensemble [15, 16]. We show that our method provides a quantitative measure of the abundance of distinct conformational states across DNA topoisomers and captures the transitions between them, offering insights into structural flexibility and variability under more physiologically relevant conditions. Such metrics are difficult to resolve with conventional techniques such as gel electrophoresis, which typically reports only a single band for each topoisomer despite the DNA adopting multiple conformations, or cryo‐ET, which yields a relatively low‐resolution snapshot of the DNA conformation upon freezing due to low signal to noise ratio [15].

We also show that Procrustes analysis of molecular splines offers a useful means of guiding feature extraction in neural networks. By providing Procrustes distances as supervisory information during ResNet18 [23] training, the network was encouraged to embed AFM crops into a feature space where conformationally similar molecules cluster together and distinct shapes are driven apart, ensuring that extracted features reflect molecular geometry rather than AFM image artifacts such as row misalignment, scanner drift or tip convolution. Furthermore, we demonstrate that our approach enables automated comparison between molecular dynamics (MD) simulations and AFM data by reducing structures from both modalities to a common spline representation. This allows conformational ensembles obtained in silico to be quantitatively compared with those observed experimentally, providing a means of assessing how well simulations capture the true structural heterogeneity of DNA.

In summary, we present an automated approach to define conformational states of DNA molecules under superhelical stress. Building on earlier observations that supercoiling drives the formation of compact, self‐crossing conformations from open circular structures, our method reveals and quantitatively resolves transitional states that connect these extremes. This achievement advances DNA structural analysis from descriptive classification to quantitative mapping of the conformational continuum and would generalize well to other applications where conformational heterogeneity underlies biomolecular function. In addition, we demonstrate how the framework enhances complementary methodologies, guiding a neural network to extract conformation‐relevant features and enable direct comparison between AFM experiments and MD simulations to understand where structural differences arise.

## 2 Methods

### 2.1 Minicircle Preparation

Plasmid pMC339‐FAR was derived from pMC339‐BbvCl [17] by the incorporation of a triplex binding site using the QuikChange site‐directed mutagenesis kit (Stratagene). This plasmid was transformed into *E. coli* strain LZ54 [24] and 339 bp minicircles were generated using bacteriophage λ‐Int site‐specific recombination as described previously [17]. The sequence of the 339 bp minicircle is listed below:

TTTATACTAACTTGAGCGAAACGGGAAGACAAACTTTCTTGTTCACCGAAACGCGCGAG
GCAGCTGTATGGCGAAATGAAAGAGTTCTTCCCGGAAAACGCGGTGGAATATTTCGTTT
CCTACTACGACTACTATCAGCCGGAAGCCTATGTACCGAGTTCGAGAGAGAGAGAGAGA
AAAGATGCCTCAGCTCTGTTACAGGTCACTAATACCATCTAAGTAGTTGATTCATAGTGAC TGCATATGTTGTGTTTTACAGTATTATGTAGTCTGTTTTTTATGCAAAATCTAATTTAATATAT TGATATTTATATCATTTTACGTTTCTCGTTCAGCTTT.

### 2.2 Generation and Purification of Minicircle Topoisomers

Minicircle DNA was first nicked at a single site using Nb.BbvCI (New England Biolabs) following the manufacturer's protocol. The nicking endonuclease was subsequently inactivated by incubating at 80°C for 20 min. Negatively supercoiled topoisomers were generated by religating the nicked minicircle in the presence of varying concentrations of ethidium bromide (EtBr) as described previously [17], or in the absence of ethidium bromide for the relaxed topoisomer (Δ*Lk* = 0). EtBr is known to underwind the DNA helix, the underwinding is maintained when the minicircle is re‐ligated, manifesting as negative supercoiling when the EtBr is extracted with butanol. Individual minicircle topoisomers, with specific ∆Lk values, were isolated by preparative polyacrylamide gel electrophoresis as described previously [14]. Briefly, more negatively supercoiled topoisomers migrate further through the gel matrix allowing for individual topoisomers to be separated. Topoisomers extracted from the gels were resuspended in 10 mM Tris pH 8.0 and DNA concentrations determined using a Nanodrop Spectrophotometer (ThermoFisher Scientific, Waltham, MA). Samples of the isolated topoisomers were analyzed on a polyacrylamide gel to ensure the topoisomer population was identified correctly and comprised a single, definitive linking number.

### 2.3 Minicircle Sample Preparation for AFM

The 339 bp DNA minicircles topoisomers, e.g., Δ*Lk* = ‐1 were diluted to 5 ng µL^−1^ using MilliQ water. 1 µL (5 ng) of DNA was immobilized on freshly cleaved muscovite mica using 25 µL of immobilization and imaging buffer (3 mm NiCl_2_ and 10 mm HEPES, pH 7.4), hereafter referred to as “adsorption buffer,” for 5 minutes at room temperature. Following this incubation, the adsorption buffer was removed and the mica was washed 4 times with fresh adsorption buffer. Post washing, 25 µL of adsorption buffer was added and the sample loaded onto the AFM. During imaging if the volume of adsorption buffer decreased due to evaporation, it was replenished with MilliQ water to minimize changes to buffer conditions. This imaging protocol was repeated for each of the 339 bp topoisomers generated, producing an AFM image dataset for each topoisomer.

### 2.4 AFM Image Acquisition

All imaging was performed in adsorption buffer. AFM was conducted using a Bruker Dimension XR (FastScan) microscope in PeakForce Tapping Mode. Bruker FastScan‐D probes were chosen for this application due to their nominal tip radius of 5 nm, spring constant of 0.25 N m^−1^, and resonant frequency of 110 KHz. Forces applied to samples varied between 50 and 150 pN depending on scan size. PeakForce amplitude was maintained at 10 nm with PeakForce tapping frequency set between 8–12 kHz. Images were recorded at a resolution of ≤ 0.5 nm per pixel at a scan rate of 2.9 to 3.3 Hz.

To ensure the tip radius did not become too large and interfere with the downstream analysis, the width of the DNA molecules was monitored during imaging (Figure S1a). If the measured DNA width increased above 6 nm (and therefore the tip width increased above 4 nm [25] (Figure S1b), the probe was changed.

### 2.5 MD Simulations

Simulations for the Δ*Lk* = 0, −1, −2, −3, and −6 topoisomers of the same 339 bp minicircle were previously performed in implicit solvent [16]. Each simulation was initiated from a perfectly planar circular conformation and allowed to equilibrate, enabling the torsional stress to redistribute between changes in the molecular twist and the emergence of coiled coil (writhe) structures. To further capture the conformational variability, the trajectories were extended for an additional 20 ns using a reduced water viscosity, thereby accelerating the relevant dynamic timescales by at least an order of magnitude. Each trajectory comprised 1000 frames.

### 2.6 Molecular Spline Generation from AFM Images

AFM images were processed using TopoStats (v2.3.1), which performs image filtering, DNA segmentation, and DNA tracing to generate single‐molecule splines that can be used for shape analysis. The details of these processing steps have previously been described in detail in Beton et al. [11] and Holmes et al. [12]. Briefly, molecule traces are iterated so the trace (and therefore the spline) follows the highest point of the DNA. This ensures that the spline follows the central line of the DNA and provides an accurate trace. This is reflected in contour length measurement of these data wherein the median trace length is 107 nm compared to the expected value of 115 nm (for 339 bp of B‐DNA) (Figure S1c).

For this analysis, a custom U‐Net segmentation model was employed to preserve intricate structural detail, such as internal holes and closely passing strands, that are often lost with standard threshold‐based segmentation [12]. A total of 78 AFM images of 339 bp DNA minicircles were manually segmented into DNA and background to generate the training dataset. To increase the number of images for training, this dataset was augmented twofold using random transformations, including up to a 30% increase in scale, up to a 30% translation, integer rotations of 90°, and horizontal or vertical flipping. This prevented the model from learning spurious correlations, i.e., overfitting wherein the model learns which images belong to a set rather than learning which features to extract as part of the analyses. All images were resized to 512 × 512 pixels before being passed into the network. The labelled dataset was randomly partitioned into training (80%) and test (20%) sets. The model was trained using the Adam optimizer with a learning rate of 0.001, a batch size of 5, 120 steps per epoch, and 100 training epochs. Binary cross‐entropy was used as the loss function. The U‐Net architecture comprised a five‐layer encoder–decoder network with skip connections between corresponding levels.

The torsional strain associated with negative supercoiling occasionally leads to disruptions to base pairing and single‐stranded regions in the 339 bp minicircles. This can often be visualized as regions of DNA that are lower than its expected 2 nm height within the AFM images, leading the U‐Net model to misclassify these areas as background. This results in molecular splines that also have gaps within them and cannot be used for Procrustes shape analysis. To address this issue, a gap‐filling algorithm was implemented to “repair” the resulting incomplete DNA splines prior to shape analysis. Open contours were identified by the Euclidean distance between their terminal points; those with a gap smaller than 20% of the expected contour length were eligible for correction. The 20% threshold, equivalent to a maximum gap of < 23 nm, was manually chosen to prevent the exclusion of fragmented splines that could be reliably reconnected. This value aligns with the characteristic defect size observed in this study and in Pyne et al. [16].

For each eligible molecule, short coordinate segments on either side of the break were extracted to provide local geometric context, and a cubic spline was then fitted through these points to generate a smooth, continuous bridge connecting the two ends. The interpolated bridge was then merged with the original contour to form a continuous, closed molecule, which was subsequently resampled to 120 evenly spaced points. A total of 248, 276, 232, 231, 227, 177, and 237 complete splines were obtained for the Δ*Lk* = 0, –1, –2, –3, –4, and –6 339 bp minicircle topoisomers, respectively.

### 2.7 Molecular Spline Generation from MD Simulations

Helical axis trajectories were extracted from MD simulations using the ReWrLINE Python package, which outputs the helical axis coordinates for each simulation frame in.xyz format. Each block of coordinates within the file corresponds to a single frame and was read into Python as a 3D contour. For each contour, points were centered at their centroid and aligned using principal component analysis to orient the major axis of variance along a common reference frame. The aligned contours were then projected onto the first two principal components, yielding a 2D representation of the DNA helical axis suitable for comparison with experimental AFM splines. Finally, each projected contour was normalized to unit scale by dividing by the maximum radial distance from the origin, ensuring consistent scaling across all 1000 frames within each MD simulation trajectory.

### 2.8 Procrustes Analysis

DNA splines from AFM images (a total of 1626 splines across all topoisomers) and from MD simulations (1000 per trajectory, with every 40th frame retained for analysis) were resampled to a fixed number of evenly spaced points (120) to enable direct comparison between molecules of different lengths. Each spline was centered and normalized to unit scale, removing differences in position and length. Because DNA circles lack a natural starting point, one spline was cyclically shifted through all possible positions and optimally aligned to the reference using Procrustes analysis, implemented with the scipy.spatial.procrustes function (SciPy v1.10, Python 3.10), which applies rotation and scaling to minimize disparity. The smallest residual sum of squared differences across all shifts was retained as the cyclic Procrustes distance, providing a quantitative measure of shape dissimilarity; small values indicate closely related conformations while larger values reflect substantial geometric differences. Pairwise distances were computed between all AFM and simulation splines to generate a dissimilarity matrix, which was subsequently used to identify and visualize the closest‐matching structures across datasets.

### 2.9 Cluster and Enrichment Analysis

Hierarchical clustering was performed directly on the cyclic Procrustes distance matrix, which quantified pairwise shape dissimilarities between DNA splines. Agglomerative clustering was carried out with an average linkage criterion (AgglomerativeClustering, scikit‐learn) using the precomputed Procrustes distances as input. Where a specific number of clusters *K* was required, this requirement was enforced directly in the clustering routine. Otherwise, we determined the optimal number of clusters by sweeping across candidate values of *K* and selecting the solution that maximized the silhouette score.

### 2.10 ResNet18 Training and Validation

A convolutional neural network was trained to learn image embeddings of AFM minicircle images that reflect pairwise molecular shape similarities derived from cyclic Procrustes distances. 1630 AFM crops were loaded in grayscale, replicated to three channels, resized to 224 × 224 pixels, converted to tensors, and normalized to zero mean and unit variance (mean = std = 0.5 per channel). A ResNet‐18 backbone (final fully connected layer removed) was used to extract image features, followed by a two‐layer fully connected head (512 → 512 → 128 with ReLU activation) producing 128D embeddings. Embeddings were L2‐normalized to unit length so that cosine similarity represented relational proximity between molecules. Training aimed to align embedding‐space similarities with the experimentally derived Procrustes distances. For each mini‐batch of 128 AFM minicircle images, the corresponding submatrix of Procrustes distances (D_B) was converted to a target similarity matrix defined as S_ij = exp[‐(D_ij / σ)^2^], with σ = 0.05. The network's cosine similarities were computed as Ŝ_ij = z_iᵀ · z_j, and compared to S_ij using mean‐squared error over off‐diagonal entries. This objective encouraged conformationally similar molecules to cluster together and dissimilar ones to separate in the learned embedding space.

The model was trained for 20 epochs using the AdamW optimizer (learning rate = 2 × 10^−^^4^, weight decay = 1 × 10^−^^4^) with batch size 128 and gradient‐norm clipping at 1.0. After each epoch, embeddings for all molecules were computed and evaluated by the Spearman correlation between cosine distances in embedding space and the corresponding Procrustes distances on a random subset of 400 molecules. The model checkpoint with the highest correlation coefficient (ρ) was retained as the best representation of shape‐consistent embeddings.

### 2.11 Conformational Subtype Classification

A supervised classification model was trained to identify DNA conformations from AFM image embeddings. The 128D embeddings obtained from the best ResNet‐18 checkpoint were paired with manually assigned class labels (circle, teardrop, and rod from figure‐8) for supervised training. Each of the four conformational classes was represented by approximately 30 images. Labelled data were split into 75% training and 25% test sets using stratified sampling to preserve class balance, and the training set was upsampled to equalize class counts. A gradient‐boosted tree model (XGBoost) was trained with 600 estimators, a maximum depth of 4, a learning rate of 0.05, subsampling and column sampling rates of 0.9, and default regularization (*λ* = 1, *α* = 0). Model performance was evaluated on the held‐out test set using per‐class precision, recall, F1‐score, and a confusion matrix. The classifier was then applied to all embeddings, and only predictions with a confidence score ≥ 0.5 were retained for downstream analysis, with lower‐confidence cases labelled “Other.” Class proportions were calculated from confident predictions (*n* = 263 for Δ*Lk* = 0, 226 for −1, 220 for −2, 221 for −3, 170 for −4, 231 for −6, and 243 for the native sample) and plotted as vertical bars showing conformational distributions across topoisomers.

### 2.12 Saliency Maps

Occlusion‐based saliency mapping was used to identify the image regions most important for distinguishing minicircle conformational classes (“Circle,” “Teardrop,” “Rod,” and “Fig8”). Each AFM image was passed through the trained convolutional neural network and downstream classifier to obtain class probabilities. To estimate the contribution of local regions, a 24 × 24 pixel patch was systematically occluded across each image, and the resulting decrease in predicted probability for the target class was recorded. This produced an occlusion heatmap in which warmer colors indicate regions whose removal most strongly reduced classification confidence, that is, the most important pixels for the model's decision. For each class, occlusion maps were generated for up to 30 classified images. To account for positional variability, each map was recentered based on the intensity‐weighted centroid of its top 5% of saliency values (≥95th percentile) before averaging. The resulting centered averages therefore highlight the spatial regions that most consistently contributed to classification across the selected subset of images. The final averaged heatmaps were overlaid on representative AFM images to illustrate the structural features most strongly used by the model to distinguish between DNA conformations.

### 2.13 Dimensionality Reduction

To visualize conformational relationships among DNA minicircles, pairwise similarity matrices derived from Procrustes were embedded into 2D space using multidimensional scaling (MDS) and uniform manifold approximation and projection (UMAP). MDS was implemented in scikit‐learn (function MDS, metric = True, n_components = 2, random_state = 42), applied directly to the precomputed distance matrix. UMAP embeddings were generated using the umap‐learn package (n_neighbors = 15, min_dist = 0.1, n_components = 2, metric = “precomputed”, random_state = 42).

### 2.14 Statistical Analyses

To assess overall conformational differences among groups of DNA minicircles (e.g., relaxed versus supercoiled or among the various topoisomers), we applied permutational multivariate analysis of variance (PERMANOVA) using 9999 permutations as implemented in scikit‐bio (function permanova), with pairwise post‐hoc comparisons and false discovery rate (FDR) correction for multiple testing. Differences in 1D summary statistics (e.g., mean Procrustes distance to the relaxed topoisomer (Δ*Lk* = 0) were assessed using the non‐parametric Kruskal–Wallis test, followed by Dunn's post‐hoc test with FDR correction. Where monotonic trends were expected (e.g., increasing divergence with supercoiling), relationships between variables (such as Δ*Lk* and distance metrics or geometric features) were quantified using Spearman rank correlation coefficients (*ρ*).

Feature‐based analyses were performed using Kruskal–Wallis tests across topoisomer states, with FDR correction to identify significantly varying features (*q* < 0.05). Features showing significant monotonic trends with Δ*Lk* (|*ρ*| ≥ 0.25) were retained for further multivariate analysis. The full list of geometric features extracted through TopoStats and used within the feature‐based analyses performed here is provided in Table S1.

Unless otherwise stated, statistical significance was defined as *p* < 0.05 after multiple‐testing correction. All values are reported as mean ± standard deviation unless otherwise specified.

## 3 Results

### 3.1 Shape Space Analysis Reveals Conformational Differences between Relaxed and Negatively Supercoiled 339 bp DNA Minicircles

**FIGURE 1:**
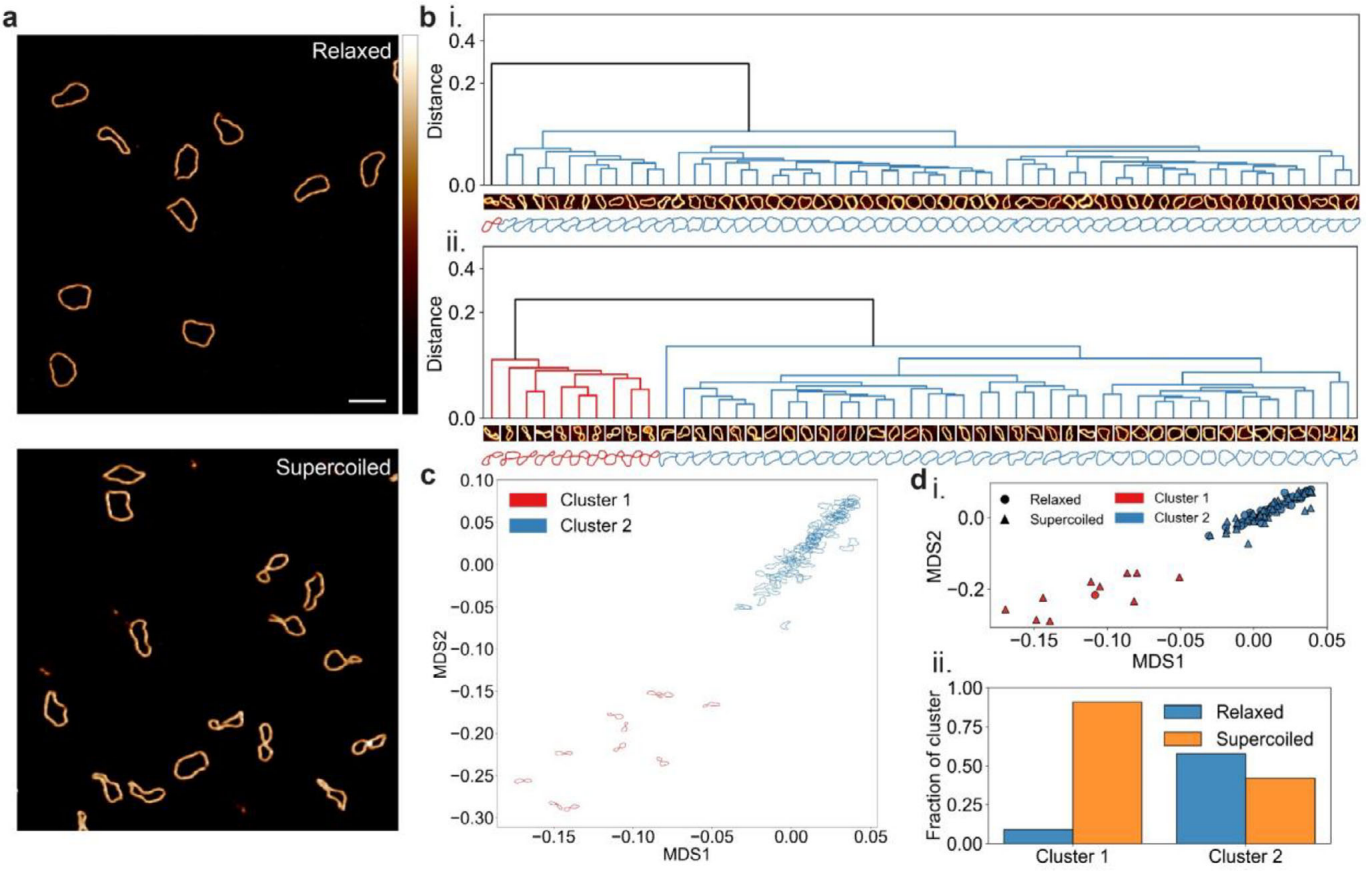
Supercoiling drives enrichment of compact, self‐crossing conformations in DNA minicircles. a) Representative AFM images of relaxed and negatively supercoiled 339 bp DNA minicircles. Scale bar = 50 nm; color scale = 0 nm to 3 nm. b) Hierarchical clustering dendrograms of the Procrustes distance matrices for relaxed (i) and supercoiled (ii) minicircles, revealing two main clusters: open conformations (blue) and self‐crossing conformations (red). To aid interpretation, both AFM images (top) and corresponding spline representations (bottom) are displayed below the dendrograms. Note that in the dendrograms, the y‐axis is shown on a square‐root scale (*y* → √*y*), which preserves the relative ordering of clusters while improving visualization for small linkage heights. c) Scatter plot of MDS embeddings of the Procrustes distance matrices for relaxed and supercoiled minicircles, illustrating the separation of the two main conformational clusters (open conformations in blue and self‐crossings in red) as well as the continuum of structures transitioning from open to compact to figure‐8 conformations. d‐i) MDS embeddings of relaxed (circles) and supercoiled (triangles) minicircles, showing the distribution of conformations across the two main clusters. Relaxed molecules primarily occupy the open cluster, whereas supercoiled molecules span both clusters, reflecting their broader conformational diversity. ii. Quantification of cluster composition, showing that Cluster 2 (open conformations) contains 55 relaxed (58%) and 40 supercoiled (42%) minicircles, whereas Cluster 1 (self‐crossing conformations) contains 10 supercoiled (91%) and 1 relaxed (9%) minicircle.

Previous studies have shown that supercoiling induces conformational changes in DNA minicircles, resulting in more compact structures with a higher incidence of self‐crossings [15, 16]. We therefore sought to validate our Procrustes shape‐space analysis method by comparing relaxed 339 bp minicircles with negatively supercoiled 339 bp minicircles. We started with the product of the initial minicircle purification process (mostly Δ*Lk* = ‐2 topoisomer), without any additional manipulation. AFM images indicated conformational changes between the relaxed and the negatively supercoiled minicircles, with relaxed samples showing more open structures and supercoiled samples exhibiting a greater number of compact, figure‐8 forms (Figure 1a). Despite these trends, heterogeneity was observed within both topoisomer samples, suggesting the presence of conformational subpopulations consistent with the intrinsic flexibility of DNA minicircles. Hierarchical clustering of the Procrustes distance matrices indicated that two clusters best described the combined data set of relaxed and supercoiled samples, corresponding to molecules with (shown in red) or without a self‐crossing (shown in blue) (Figure 1b). The dendrograms within Figure 1b also highlight the conformational landscape of both samples, spanning open circles, teardrops (because AFM images cannot be viewed from different angles like our previous cryo‐ET images where this shape was referred to as an “open figure‐8” [15], here we can only describe what it looks like from the AFM image), compact (“needle”) conformations, and figure‐8 structures. We also tested different values for the number of clusters to be considered by hierarchical clustering, *K* to see whether additional conformational subgroups can be observed. In this case, we assessed cluster numbers *K* = 3, 4, and 5. With *K* = 3, a single molecule formed its own cluster, with *K* = 4 also resulting in an additional molecule forming its own cluster. In contrast, *K* = 5 enabled identification of an additional cluster that subset the original cluster of open minicircles into two groups, with one consisting of more compact conformations (Figure S2).

The dynamic conformational landscape of DNA minicircles is more intuitively visualized in the plot of multidimensional scaling (MDS) embeddings Figure 1c, where the continuum of conformations and separation among the clusters is evident. Notably, the figure‐8 conformations (Cluster 1) occupy a more dispersed region of the embedding space, reflecting their great structural heterogeneity. In contrast, the non‐crossing conformations (Cluster 2) cluster more tightly, indicating more uniformity in their geometry. Figure 1d illustrates the proportional split between relaxed and supercoiled minicircles across the two clusters. While Cluster 2 (open, non‐crossing conformations) is enriched in relaxed molecules (making up 58% of the relaxed and 42% supercoiled), Cluster 1 (self‐crossing figure‐8 conformations) is what the negatively supercoiled topoisomer predominantly comprises (91%) compared to relaxed (9%). These distributions suggest that supercoiling promotes access to a wider spectrum of conformations, spanning both the open and the crossed states, whereas relaxed molecules are largely confined to open forms.

### 3.2 Increased Supercoiling Induces Increased Conformational Changes in 339 bp DNA Minicircles

**FIGURE 2:**
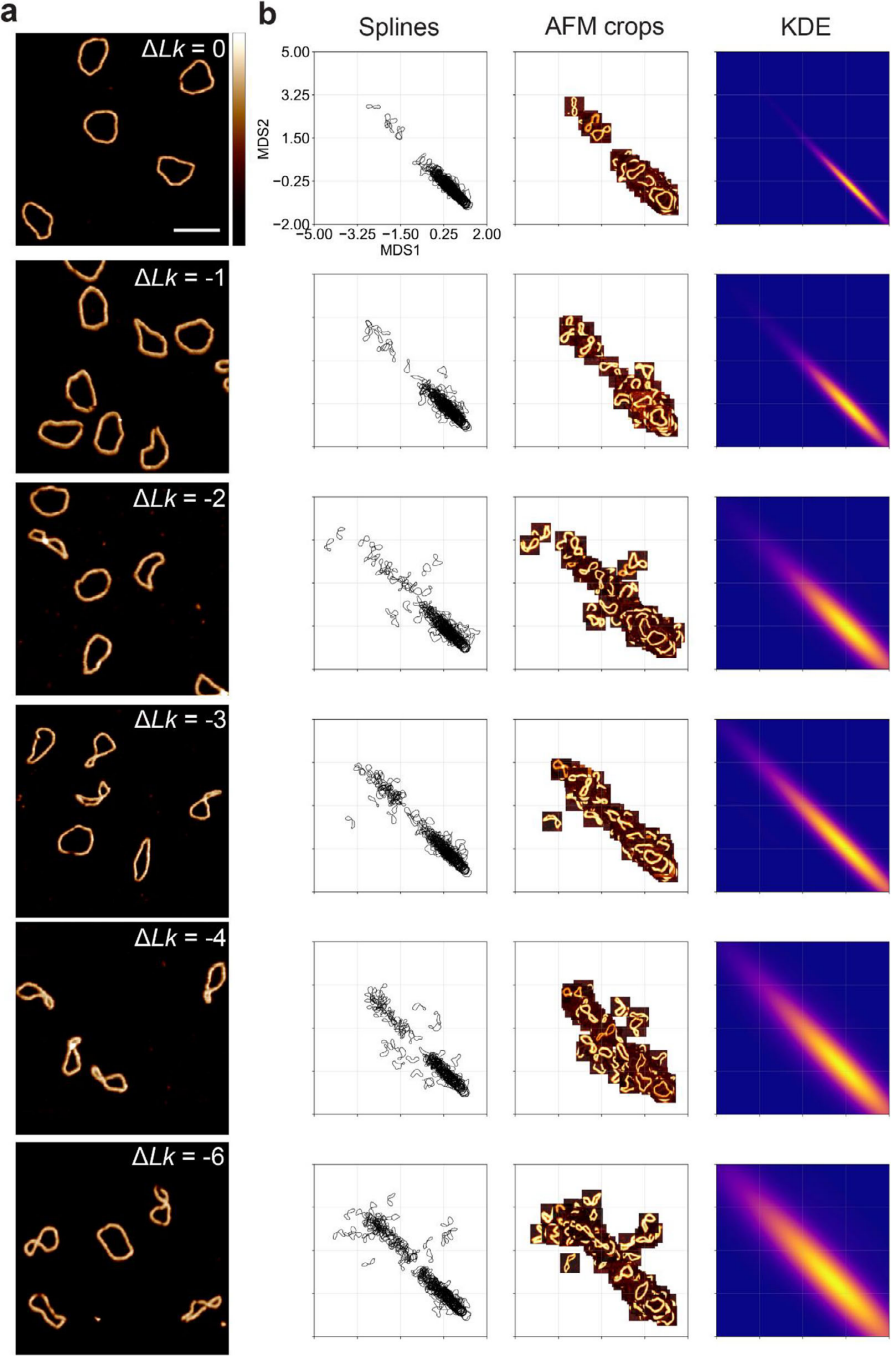
Conformational diversity of 339 bp DNA minicircles increases with increasing negative supercoiling. a) Representative AFM images of 339 bp DNA minicircle Δ*Lk* = 0, −1, −2, −3, −4, and −6 topoisomers, highlighting the increasing conformational heterogeneity with negative supercoiling. Scale bar: 50 nm; color scale: 0 to 3 nm. b) MDS embeddings of the shared Procrustes distance matrix reveal the conformational landscape across all topoisomers as a function of Δ*Lk*. Left: spline overlays demonstrate clustering of relaxed (Δ*Lk* = 0 minicircle topoisomer) and increasing dispersion with increasing negative supercoiling. Middle: corresponding AFM images are positioned in the embedding space. Right: KDE plots highlight the shift from a single concentrated hotspot (for the Δ*Lk* = 0 minicircle topoisomer) to progressively flatter, more extended distributions as negative supercoiling increases. The MDS plots for: Splines, AFM crops and KDE within the grid share the same axes as in the first MDS plot.

Having validated our shape space analysis approach by comparing relaxed and natively supercoiled minicircles, we next asked whether the method could resolve more subtle differences between topoisomers. We therefore examined relaxed 339 bp DNA minicircles (Δ*Lk* = 0) alongside a series of progressively more negatively supercoiled states (Δ*Lk* = −1 to −6). Representative AFM images of each topoisomer provided in Figure 2a show that, by eye, relaxed minicircles (Δ*Lk* = 0) appear predominantly open and circular, whereas progressively more negatively supercoiled topoisomers (Δ*Lk* = −1 to −6) increasingly adopt compact conformations, often featuring self‐crossings and figure‐8 structures.

Calculating a shared Procrustes distance matrix, across all topoisomer groups, and projecting the results into MDS space revealed how the conformational landscape changes with supercoiling (Figure 2b). Relaxed minicircles (Δ*Lk* = 0) clustered tightly in a small, well‐defined region, reflecting their structural homogeneity. In contrast, progressively more negatively supercoiled topoisomers (Δ*Lk* = −1 to −6) occupied increasingly dispersed regions of shape space, with Δ*Lk* = −6 covering the broadest span of conformations. This structural heterogeneity likely reflects a balance between molecules that relieve torsional stress through localized defect formation, yielding more open and relaxed conformations, and others that accommodate it through increased writhe and compaction, forming figure‐of‐eight structures. The KDE density plots in (Figure 2b) further highlight this progression: Δ*Lk* = 0 showed a sharp, localized hotspot, indicating that most molecules converged on a single open conformation. With increasing negative supercoiling, the density peak progressively broadened and flattened, producing more diffuse bands across the embedding. By Δ*Lk* = −6, the distribution no longer exhibited a single strong maximum but instead spread into an extended region of moderate density, consistent with molecules populating a wider range of conformations.

These visual observations were supported by quantitative analysis of the MDS embeddings. Variance in MDS1 and MDS2 reflects the average spread of conformations around the centroid of each sample, while the convex hull area provides a measure of the total extent of shape space occupied, and the KDE peak density indicates whether molecules are concentrated into a single dominant state or dispersed across multiple states. Relaxed minicircles (Δ*Lk* = 0) showed the lowest variance (0.53) and smallest convex hull area (1.76), consistent with their tight clustering, and had the highest KDE peak density (7.65), demonstrating that the majority of molecules converged on a single open conformation. Δ*Lk* = ‐1 displayed slightly greater heterogeneity, with variance of 0.90 and a convex hull of 5.04, but still retained a relatively sharp KDE peak of 3.16. Δ*Lk* = ‐2 expanded considerably in shape space (variance 1.58, hull 12.55) and showed a lower KDE peak of 1.62, indicating a broader distribution of conformations. Δ*Lk* = ‐3 and Δ*Lk* = ‐4 occupied similar intermediate regions, with variances of 2.13 and 2.25, hull areas of 8.38 and 8.52, and KDE peaks of 1.65 and 1.07 respectively, reflecting a balance between clustering and dispersion. Finally, the most negatively supercoiled state, Δ*Lk* = −6, displayed the greatest heterogeneity, with variance of 3.02, convex hull area of 13.79, and the lowest KDE peak density of 0.71.

To formally test whether topoisomers differed in their conformational distributions, we performed a PERMANOVA on the Procrustes distance matrix (Table S2). The global test revealed highly significant overall differences between groups (pseudo‐F = 43.9, *p* = 0.0001, 9999 permutations), demonstrating that changes in Δ*Lk* are associated with systematic shifts in DNA minicircle conformations. Pairwise comparisons identified the specific sources of variation. The relaxed state (Δ*Lk* = 0) was significantly different from all supercoiled states (Δ*Lk* = −1 to −6; all *q* < 0.002), confirming that relaxation produces a distinct, homogeneous conformational ensemble. In this context *q* is an adjusted p‐value correcting for the false discovery rate (FDR) that can occur from multiple comparison testing. Likewise, the most negatively supercoiled molecules (Δ*Lk* = −6) were significantly different from every other topoisomer (all *q* < 0.0003), consistent with their markedly broader and more heterogeneous conformational landscape. Intermediate states exhibited progressive differentiation from one another. Δ*Lk* = −1 differed significantly from Δ*Lk* = −2, −3, and −4, while Δ*Lk* = −2 and Δ*Lk* = −3 were also distinct from one another (*q* < 0.02). The only non‐significant comparison was between Δ*Lk* = −3 and Δ*Lk* = −4 (*q* = 0.15), indicating that these adjacent topoisomers occupy overlapping regions of shape space.

Together, these results demonstrate that relaxed minicircles are confined to a narrow, homogeneous region of shape space, while increasing negative supercoiling progressively broadens the conformational landscape, ultimately generating a highly diverse ensemble of compact and self‐crossing conformations.

### 3.3 Feature‐ and Shape‐Based Analyses Reveal Consistent Supercoiling‐Induced Structural Divergence from Relaxed Minicircles

**FIGURE 3:**
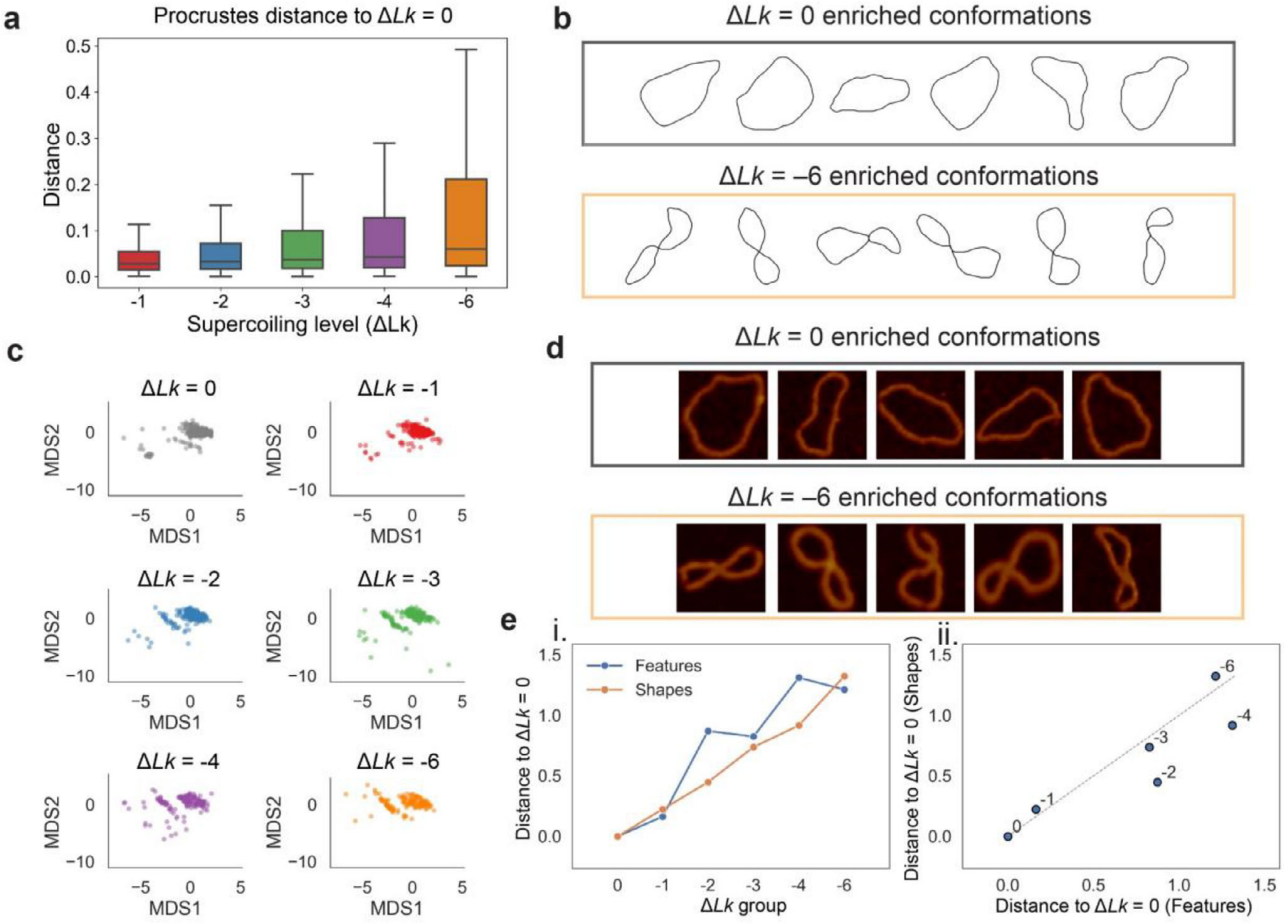
Negative supercoiling drives systematic conformational divergence captured by both shape‐space and geometric feature analyses. a) Procrustes distance of each topoisomer (Δ*Lk* = −1 to −6) to the relaxed state (Δ*Lk* = 0). Boxplots show median, interquartile range, and whiskers (1.5× IQR). Distances progressively increase with negative supercoiling, reflecting systematic divergence away from relaxed conformations. The ± errors in the text reflect the true error values for each group, the negative values have been limited to 0.0 by the axis as the Procrustes distance cannot be negative. b) Representative splines from the two conformational clusters identified by hierarchical clustering in shape space. Cluster 1 (top) is enriched in Δ*Lk* = 0 molecules and is characterized by open, circular conformations. Cluster 2 (bottom) is enriched in Δ*Lk* = −6 molecules and displays self‐crossing conformations. c) MDS embeddings of geometric features extracted using TopoStats, faceted by Δ*Lk*. Each point corresponds to an individual DNA minicircle. Embeddings are based on the four non‐redundant features retained after screening (total branch lengths, mean radius, number of crossings, and minimum Feret distance), illustrating how feature space varies with supercoiling level. d) Representative AFM crops from the two conformational clusters identified by hierarchical clustering in geometric feature space. Although derived independently of the shape‐based analysis, the same broad conformational classes emerged as in (b). e) i. Distances to Δ*Lk* = 0 increase progressively with negative supercoiling for both feature‐based (blue) and Procrustes‐based (orange) analyses. ii. Scatter plot showing strong correlation between feature‐ and shape‐based distances across the different minicircle topoisomers (Pearson *r* = 0.90, *p* = 0.014), demonstrating that conventional geometric descriptors capture the same supercoiling‐induced divergence as shape‐space analysis.

We quantified the extent to which each topoisomer diverged from the relaxed Δ*Lk* = 0 minicircle conformational ensemble. Procrustes distance analysis (Figure 3a) revealed a progressive increase in deviation with increasing negative supercoiling, demonstrating that the accumulation of torsional stress drives systematic conformational shifts away from the relaxed state. This shift was reflected in the group mean distances from Δ*Lk* = 0, which increased stepwise from Δ*Lk* = −1 (0.0525 ± 0.0726) to Δ*Lk* = −2 (0.0686 ± 0.0912), Δ*Lk* = −3 (0.0858 ± 0.1065), Δ*Lk* = −4 (0.0957 ± 0.1117), and reached a maximum for the Δ*Lk* = −6 minicircle topoisomer (0.1248 ± 0.1299).

A Kruskal–Wallis test across topoisomers (*H* = 1.38 × 10^4^, *p* < 0.0001) revealed that the distributions of distances for the negatively supercoiled topoisomers (Δ*Lk* = −1 to ‐6) compared to the relaxed (Δ*Lk* = 0) differed significantly. Dunn post‐hoc tests with false discovery rate correction further showed that every possible pairwise comparison, even among the different negatively supercoiled topoisomers was highly significant (all *q* < 1 × 10^−^^6^ ^8^), indicating that each incremental increase in negative supercoiling produces a measurable conformational divergence from the relaxed baseline. Together, these results highlight not only the overall effect of superhelical stress on the distribution of conformations but also the graded nature of conformational change across topoisomeric states.

To better understand the nature of the conformational differences identified through distance and embedding analyses, we next performed hierarchical clustering on the Procrustes distance matrix. This approach groups molecules according to overall shape similarity, enabling us to identify distinct conformational groups rather than only quantifying their spread in shape space.

Inputting all the collected AFM minicircle shapes for all topoisomers (total *n* = 1380), this analysis initially resulted in two major clusters. Cluster 1 (containing *n* = 1177 different minicircles) was the largest cluster and contained predominantly open conformations. As such, it was enriched (23%) for relaxed (Δ*Lk* = 0) topoisomer at 270 molecules, Δ*Lk* = −1 with 221 (19%), Δ*Lk* = −2 with 205 (17%), Δ*Lk* = −3 with 185 (16%), Δ*Lk* = −6 with 161 (14%), and Δ*Lk* = −4 with 135 (12%) molecules.

Cluster 2 (*n* = 203) comprised more compact, self‐crossing conformations and was enriched in highly supercoiled topoisomers. Here, Δ*Lk* = −6 contributed 76 molecules (37%), Δ*Lk* = −3 and Δ*Lk* = −4 each contributed 42 (21%), Δ*Lk* = −2 contributed 26 (13%), Δ*Lk* = −1 contributed 11 (5%), and Δ*Lk* = 0 even contributed 6 (3%) molecules.

Figure 3b illustrates representative spline examples from Cluster 1 and Cluster 2 to highlight their contrasting conformations. The majority of relaxed molecules (Δ*Lk* = 0) fell into the open cluster, with 98% assigned to Cluster 1 and 2% to Cluster 2. In comparison, highly supercoiled molecules (Δ*Lk* = −6) showed a broader distribution. Whereas 68% were found in the open cluster, 32% shifted into the compact, self‐crossing cluster. This demonstrates that clustering captures the known fact that relaxed topoisomers are mostly open conformations and that negative supercoiling increases the prevalence of compact, self‐crossing structures.

Having established that clustering in shape space resolves two broad conformational groups enriched in relaxed and highly supercoiled molecules, we next asked whether these differences could also be captured by conventional geometric features extracted directly from the splines. We began with 27 geometric features extracted using TopoStats [11] (see Methods). Each feature was tested for differences across topoisomers using a Kruskal–Wallis test. Of these, 24 features showed significant differences after false discovery rate correction (*q* < 0.05). To focus on systematic trends with supercoiling, we next assessed whether each feature exhibited a monotonic relationship with Δ*Lk* using Spearman correlation. Ten features passed this additional criterion (|*ρ*| ≥ 0.25). Finally, to avoid redundancy due to correlations between features, we applied correlation pruning, yielding four independent, non‐redundant descriptors: total branch lengths, mean radius, number of crossings and minimum Feret distance.

Scatter plots of the MDS embeddings derived from these four features, faceted by Δ*Lk*, are shown in Figure 3c. To test whether conventional geometric descriptors captured the same conformational trends revealed by Procrustes analysis, we next quantified variance, convex hull area, and kernel density peak height within the feature‐based MDS embeddings. Relaxed minicircles (Δ*Lk* = 0) occupied a relatively compact region of feature space, with variance of 3.15 and a hull area of 37.6, and showed the highest KDE peak density (0.308), indicating that most molecules converged on a consistent open conformation. Similarly, Δ*Lk* = −1 remained tightly clustered (variance 2.75; hull area 30.3; KDE peak 0.245). In comparison, more negatively supercoiled minicircles were substantially more dispersed. Δ*Lk* = −2 exhibited modest heterogeneity (variance 3.22; hull area 39.6; KDE peak 0.173), whereas Δ*Lk* = −3 and Δ*Lk* = −4 displayed the greatest expansion, with variances of 4.89 and 5.09 and hull areas of 73.8 and 47.5, respectively. These states also showed much flatter KDE peaks (0.130 and 0.103), consistent with molecules spreading across a wide range of conformations rather than clustering into a single dominant shape.

The most negatively supercoiled state, Δ*Lk* = −6, also occupied a broad region (variance 3.89; hull area 38.6) with a very low KDE peak (0.122), consistent with extensive conformational heterogeneity.

To assess whether geometric descriptors also partitioned the data into distinct conformational groups, we performed clustering on the feature‐based MDS embeddings. As with the Procrustes analysis, the molecules segregated into two broad clusters. Cluster 1 (n = 909) was larger and enriched in relaxed and mildly supercoiled molecules. It contained Δ*Lk* = 0 with 247 molecules (27%), Δ*Lk* = −1 with 187 molecules (21%), Δ*Lk* = −2 with 146 molecules (16%), Δ*Lk* = −3 with 126 molecules (14%), Δ*Lk* = −4 with 107 molecules (12%), and Δ*Lk* = −6 with 96 molecules (11%). Cluster 2 (*n* = 243) was smaller and composed primarily of more negatively supercoiled states. It comprised Δ*Lk* = −3 with 53 molecules (22%), Δ*Lk* = −4 with 50 molecules (21%), Δ*Lk* = −6 with 50 molecules (21%), Δ*Lk* = −2 with 35 molecules (14%), Δ*Lk* = 0 with 33 molecules (14%), and Δ*Lk* = −1 with 22 molecules (9%). Figure 3d illustrates representative spline examples from Cluster 1 and Cluster 2 to highlight their contrasting conformations.

To directly compare shape‐based and feature‐based analyses, we quantified distances from each topoisomer to the relaxed sample (Δ*Lk* = 0) using both approaches (Figure 3e). Both methods revealed the same overarching trend: distances increased progressively with increasing negative supercoiling, demonstrating that torsional stress drives systematic divergence from the relaxed state regardless of whether conformations are assessed in full shape space or reduced to geometric descriptors (Figure 3e‐i).

Furthermore, feature‐based and shape‐based distances were strongly correlated (Pearson *r* = 0.9, *p* = 0.014), indicating that conventional geometric descriptors capture the same progressive divergence from the relaxed state as the shape‐space analysis (Figure 3e‐ii). This concordance demonstrates that both approaches reflect the same underlying conformational shifts, providing methodological validation and biological interpretability.

### 3.4 Shape‐Space Analysis Informs Neural Network Training for AFM Image Classification

Using spline‐based Procrustes distances, we obtained a reliable metric of shape similarity whereby molecules with comparable geometry aligned closely whilst distinct conformations separated. Although effective, this approach becomes limiting for large datasets, as it relies on preprocessing to generate accurate spline representations and requires calculating pairwise Procrustes distances across all splines. An efficient alternative is the use of neural networks, which can learn directly from AFM crops without spline extraction or comparison, thereby accelerating analysis and reducing the amount of preprocessing required on large datasets. However, in their standard form, neural networks lack explicit awareness of the relational structure among molecular conformations. In the context of AFM data, this lack means they may prioritize superficial image features such as scan artefacts, local intensity fluctuations, or background noise, rather than those that describe molecular geometry. To address this potential issue, we incorporated information from spline‐based Procrustes distances into the training of a ResNet18 network, which was tasked with extracting features from single‐molecule AFM crops of 339 bp minicircles at different Δ*Lk* values. By using this matrix to supervise the learning process, the network is encouraged to embed AFM crops into a representation space that preserves these known shape relationships. This ensures that molecules with similar geometries are placed close together in the learned embedding, while dissimilar conformations are separated, enabling the model to learn features that are biologically meaningful rather than artifactual.

**FIGURE 4:**
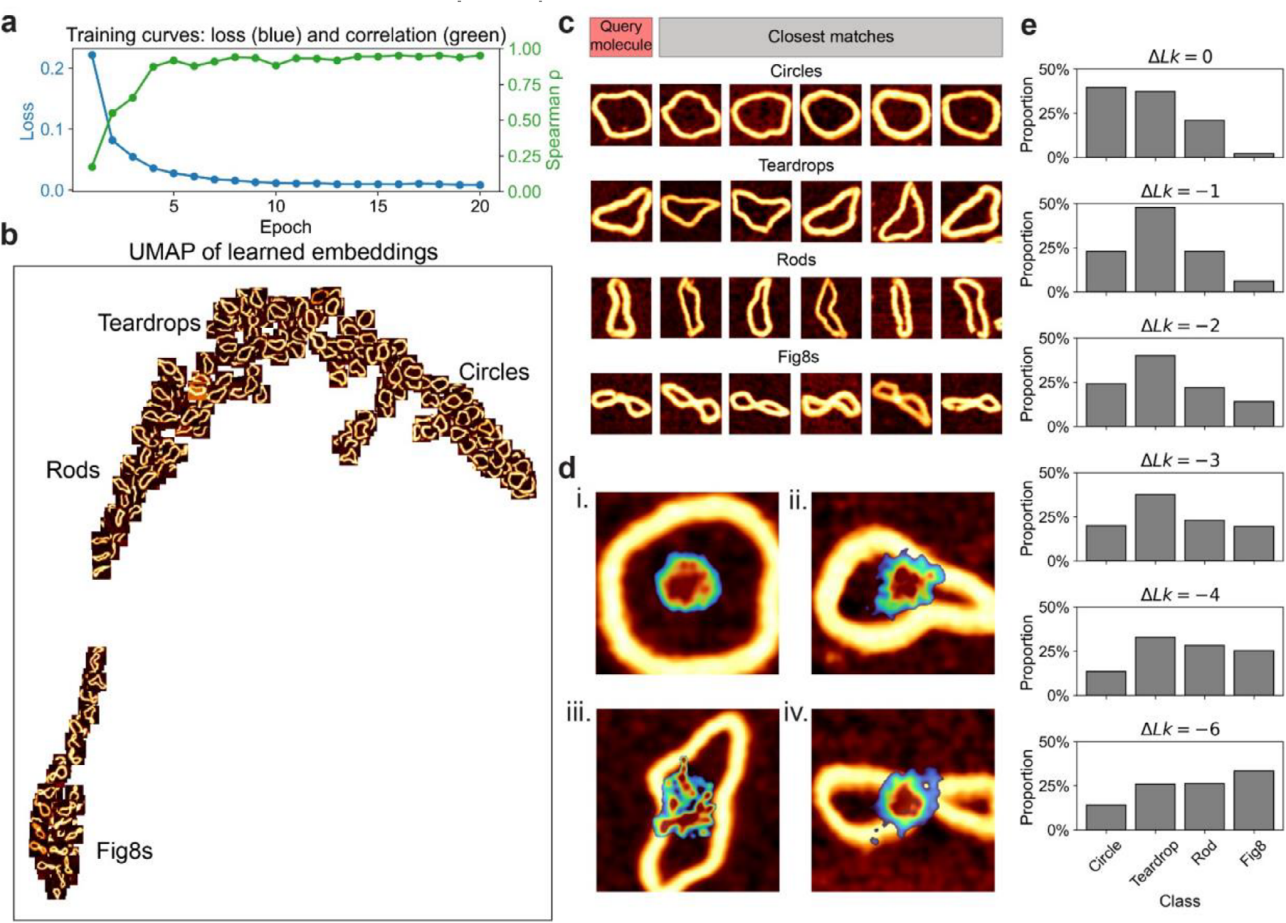
Deep learning predictions capture negative supercoiling‐dependent variance of DNA conformations. a) Training dynamics of the ResNet18 embedding network supervised by spline‐based Procrustes distances. The training loss steadily decreased across epochs, while the correlation between learned embedding distances and Procrustes distances increased from *ρ* = 0.17 to *ρ* = 0.95, indicating that the network successfully recovered the relational structure of molecular conformations. b) UMAP projection of learned embeddings. Molecules formed a continuous trajectory from circles (symmetric loops with wide openings) through Teardrops (asymmetric loops tapering to a tip) and rods (elongated, narrow loops), to figure‐8s (self‐crossing conformations), reflecting the expected progression with increasing supercoiling. c) Nearest‐neighbor analysis of embeddings. For representative query molecules from each class, the five closest neighbors retrieved in embedding space showed highly similar conformations, demonstrating that the embeddings capture biologically relevant shape features. d) Saliency maps of XGBoost classifier predictions. For each class, occlusion‐based heatmaps (averaged over 100 molecules, top 10% pixels shown) highlight the regions most important for classification. The model focused on hallmark structural features: i. the central opening in Circles, ii. tapered ends of Teardrops, iii. elongated axes of rods, and iv. crossing regions of figure‐8s. e) Class distributions across Δ*Lk* groups, based on XGBoost classification. At Δ*Lk* = 0, circles (40%) and teardrops (37%) predominated, with fewer rods (21%) and figure‐8s (2%). With increasing negative supercoiling, circles steadily declined, while figure‐8s rose to 25% at Δ*Lk* = ‐4 and 33% at Δ*Lk* = ‐6.

To guide the network, we turned the Procrustes distances into a simple similarity score between 0 and 1. In this scale, molecules that are very alike score close to 1, and molecules that look very different score close to 0. The network's task during training was to make its own embeddings agree with these similarity scores. In other words, if two molecules had a low Procrustes distance, the network was encouraged to place them close together, and if they had a high distance, it was encouraged to keep them apart. The training loss measures how well the network succeeds at this: a high loss means the embeddings don't yet reflect the Procrustes relationships, while a low loss means they do. The loss began relatively high (0.22 at epoch 1) and fell steadily to a very low value (0.008 by the final epoch), showing that the network had learned to organize the AFM crops in a way that reflects the known similarities and differences in molecular shape (Figure 4a). Alongside the loss, we also checked whether the network's learned distances between embeddings matched the original Procrustes distances by calculating their Spearman correlation. At the start of training, this correlation was very weak (*ρ* ≈ 0.17 after the first epoch), showing that the embeddings did not yet capture meaningful shape relationships. As training progressed, the correlation increased sharply, reaching *ρ* ≈ 0.87 by epoch 4, *ρ* ≈ 0.92 by epoch 5, and peaking at *ρ* ≈ 0.95 by epochs 16–18. This steady rise demonstrates that the embedding space learned by the network closely reproduced the relational structure of the molecules defined by spline analysis.

To check whether the embeddings captured meaningful aspects of DNA conformation, we visualized them using UMAP (Figure 4b). This projection revealed a clear trajectory across the embedding space: molecules arranged from open circles (symmetric loops with a wide central opening) at the top right, through intermediate teardrops (asymmetric loops that taper to a tip) and rods (elongated structures with narrow internal regions), before reaching figure‐8 (Fig. 8) conformations (characterized by a self‐crossing) at the bottom left. This continuous ordering shows that the network embeddings organized the AFM crops according to the gradual structural transitions expected from DNA supercoiling. To further evaluate the quality of the learned embeddings, we examined the five nearest neighbors of example query molecules, selecting one query molecule from each conformational class (circles, teardrops, rods, figure‐8s) (Figure 4c). Neighbors were identified based on distances in the learned embedding space, such that molecules closer in the embedding were considered more similar. In each case, the embedding space retrieved molecules that closely resembled the query, confirming that it reliably captured structural features characteristic of each class.

To assess whether the learned embeddings could support supervised classification, we trained an XGBoost classifier on the four identified conformational classes using manual labelling of 124 AFM crops (31 circles, 31 teardrops, 32 rods and 30 figure‐8s). After splitting the manually labelled set into 75% training and 25% test data, the model achieved an overall accuracy of 87% on the held‐out test set (Figure S3). Precision and recall were high across classes (circles: precision = 100%, recall = 88%; teardrops: precision = 86%, recall = 75%; rods: precision = 78%, recall = 88%; figure‐8: precision = 88%, recall = 100%). To understand which areas of the AFM crops the classifier relied on, we generated saliency maps by averaging over 100 molecules per class. This produced class‐specific heatmaps highlighting the regions most strongly associated with each prediction. The resulting patterns showed that the model consistently focused on structural hallmarks of each conformation: the open central void of circles, the narrowing tip of teardrops, the elongated axis of rods, and the central crossing of figure‐8s (Figure 4d).

Repeated stratified 5‐fold cross‐validation (20 repeats; 100 total model fits) yielded a macro‐F1 score of 0.82 ± 0.06 (mean ± SD), demonstrating consistent classification performance across data partitions. Fig8 conformations were classified with high precision and recall (F1 = 0.95), consistent with their characteristic crossing regions that generate a compact, well‐separated cluster in embedding space. In comparison, teardrop conformations showed lower recall (F1 = 0.69), reflecting their broader geometric variability and similarity with other conformations (circles and rods). This is further supported by the observed overlap of these three classes in embedding space, which contributes to increased classification ambiguity. The full table of cross‐validation performance metrics is provided in Table S3.

By applying the trained classifier to the full dataset, we obtained class distributions for each Δ*Lk* group, allowing us to track how the balance of circles, teardrops, rods, and figure‐8s shifted with increasing supercoiling (Figure 4e, Table S4). At Δ*Lk* = 0, the population was dominated by circles (40%) and teardrops (37%), with only a minority of rods (21%) and figure‐8s (2%). As Δ*Lk* decreased, circles steadily declined while teardrops remained common up to Δ*Lk* = ‐3. At higher supercoiling (Δ*Lk* ‐4 and ‐6), figure‐8s increased sharply to 25% and 33% respectively, alongside a rise in rods, reflecting the expected transition toward more compact and self‐crossing conformations, as previously described in Irobalieva et al. [15]. Teardrops and rods remained relatively abundant across all supercoiling states, reflecting their roles as intermediate conformations. In contrast, Circles were enriched under low or no supercoiling (Δ*Lk* = 0, ‐1), while figure‐8s became increasingly frequent at higher negative supercoiling (Δ*Lk* = ‐4, ‐6), representing the two extremes of the conformational trajectory.

### 3.5 Shape Space Analysis Enables Cross‐Validation of Atomistic MD Simulations and AFM

**FIGURE 5:**
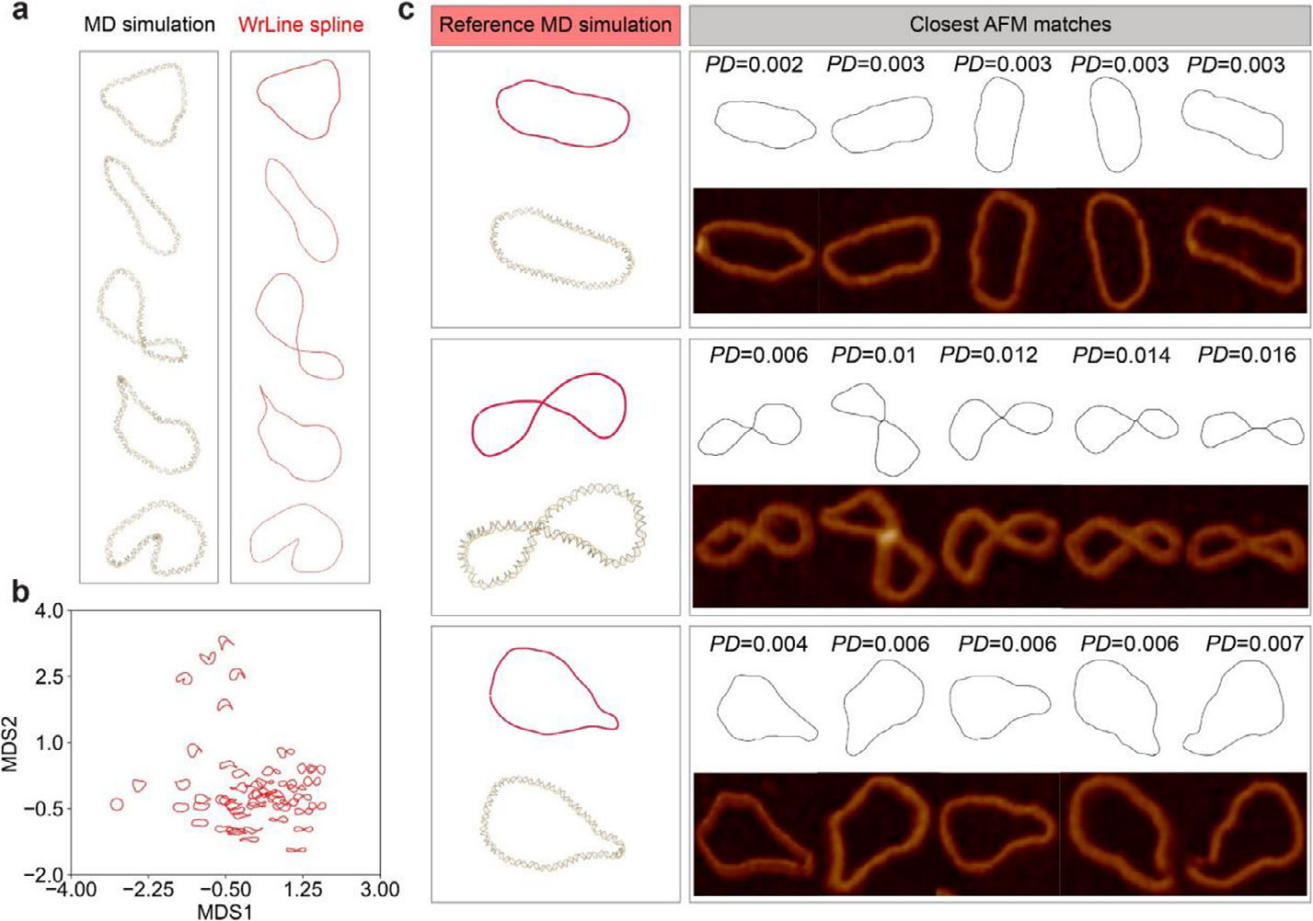
Common shape‐space framework reveals correspondence between AFM and MD conformations. a) Representative snapshots from all‐atom MD simulations of 339 bp DNA minicircles (left panel) alongside their spline representations generated with WrLINE (right panel). b) MDS embedding of Procrustes distances for the MD trajectory at Δ*Lk* = −1, resolving the sampled conformations into distinct states including open circles, teardrops, rod‐like, figure‐8, and lily pad shapes. c) Combined Procrustes analysis of AFM and MD splines. Three representative MD conformations (top: rod‐like, middle: figure‐8, bottom: teardrop) were selected as references, and their closest AFM matches are shown in ascending order of Procrustes distance (PD). The consistently small distances demonstrate quantitative alignment between simulated and experimental structures, moving beyond purely qualitative visual similarity.

Molecular dynamics (MD) simulations are a complementary tool for AFM, often used to interpret AFM findings by revealing the underlying parameters and forces that drive the conformations observed experimentally. However, because AFM images and MD simulations occupy different domains, direct structural comparison is not straightforward and is typically limited to qualitative, visual assessments of similarity. To enable more direct comparison, we used WrLINE [26], a tool that reduces all‐atom MD simulations down to their molecular backbone, yielding spline‐like representations analogous to those extracted from AFM images. This common representation allows us to apply the same shape‐space analysis pipeline to both experimental and simulated data, providing a direct framework for comparison. Figure 5a provides representative snapshots from MD simulations of 339 bp minicircles alongside their molecular splines obtained through WrLINE, and Figure 5b presents MDS embeddings of Procrustes distances for the MD trajectory at Δ*Lk* = −1, illustrating how the conformational landscape sampled in simulations can be visualized within the same shape‐space framework used for AFM data. The embedding resolves the trajectory into distinct conformational states, including open circles, teardrops, rod‐like, figure‐8, and lily‐pad shapes.

By combining AFM and MD splines into a single Procrustes distance matrix, we were able to directly compare simulated and experimental conformations within the same shape space. This made it possible to assess whether the conformational states sampled in simulations are also present within the experimental data set. Using this framework, we selected reference MD conformations and identified their closest structural matches among the AFM dataset, marked as the AFM molecules with the shortest Procrustes distance to the reference MD. Figure 5c illustrates this for three representative simulation frames (rod‐like, figure‐8, and teardrop), with AFM molecules displayed in ascending order of their Procrustes distance from the reference MD structure. In all three cases, the closest matches corresponded to very small distances (all ≤ 0.03), showing quantitative rather than purely qualitative agreement between the AFM and MD conformations. The lily‐pad conformation observed in simulations was not identified in the AFM dataset. This likely reflects two factors: first, the projection‐picking procedure used here for simulations selects 2D views that maximize variability in coordinate space, which may bias the resulting 3D projection toward an orientation that is not representative of AFM imaging; and second, adsorption to the mica surface during AFM can restrict out‐of‐plane flexibility.

### 3.6 Quantifying Coverage of Experimental Conformations in MD Shape Space

Now that we can directly compare MD and AFM conformations within a common shape‐space framework, we can begin to assess how well simulation trajectories reflect what is observed experimentally. In particular, this approach allows us to determine which simulated conformations are well represented in the AFM dataset, which are only partially represented, and which are absent altogether.

**FIGURE 6:**
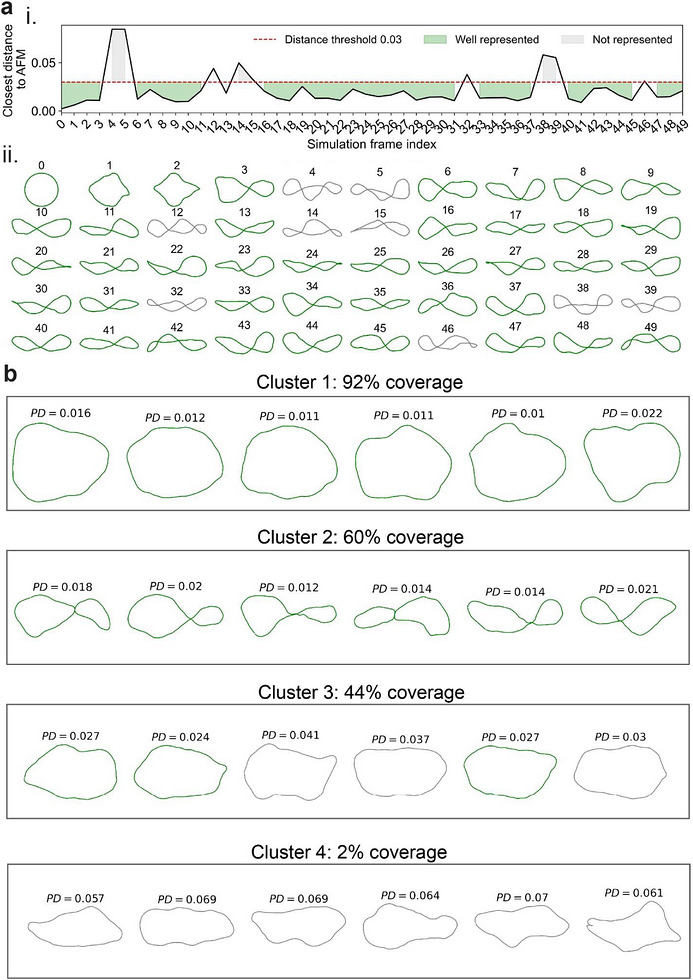
Evaluating how well MD simulations represent the experimental conformational landscape of Δ*Lk* = −2 339 bp minicircles. (a) i. For each frame in the Δ*Lk* = −2 MD trajectory, the minimum Procrustes distance to the AFM Δ*Lk* = −2 dataset was calculated. The coverage plot shows which MD frames are represented within the AFM dataset (Procrustes distance (*PD*) ≤ 0.03, colored green) and which are not (Procrustes distance > 0.03, colored gray). ii. The MD trajectory visualized frame by frame, with green frames indicating conformations represented within the AFM dataset (*PD* ≤ 0.03) and grey frames indicating conformations not captured experimentally. (b) AFM splines at Δ*Lk* = −2 clustered into four groups (K = 4) based on their distance profiles to the MD trajectory.

For each frame in the Δ*Lk* = −2 MD trajectory, we measured the Procrustes distance to its closest AFM spline within the Δ*Lk* = −2 data set. We selected the Δ*Lk* = −2 MD trajectory as a validation case, as by visual inspection it appeared to show both agreement and disagreement with AFM data, providing an opportunity to test which simulated conformations are well represented experimentally and which are absent. As shown in Figure 6a‐i, most frames had at least one experimental counterpart within a very small distance (threshold 0.03), indicating strong agreement between simulation and experiment. Frames above this threshold correspond to conformations not represented in the AFM dataset. Figure 6a‐ii visualizes this mapping across the MD simulation trajectory, with green frames denoting conformations that are represented within the AFM data and grey frames denoting those that were not. Notably, the MD conformations that were not represented within the AFM dataset corresponded primarily to molecules with two self‐crossings (frames 4, 5, 12, 14, 32, 38, 39, and 46), as such structures were absent from our experimental observations. Two frames with self‐crossings (frames 42 and 49) did fall within the 0.03 distance threshold, but their closest AFM counterparts were figure‐8 conformations that shared a broadly similar global geometry while differing in precise topological detail (Figure S4). The other exception involves the structures at the beginning of the trajectory, where the simulation had not yet equilibrated from its initial artificial, perfectly circular shape.

It is also beneficial to identify which conformations within the AFM dataset are already well represented by MD simulations, and which ones are under‐represented and therefore need to be better captured in order for MD trajectories to more faithfully reflect the conformations observed experimentally. To assess how well AFM conformations were represented in the Δ*Lk* = −2 simulation, we clustered AFM splines based on their full distance profiles to the MD trajectory. Here, if two AFM molecules both found their best matches among similar frames of the trajectory, they were placed in the same cluster, whereas molecules that aligned with entirely different parts of the simulation fell into separate clusters. Using k‐means (*K* = 4), the AFM dataset partitioned into four such clusters (Figure 6b). Cluster 1 comprised open, near‐circular molecules, whereas Cluster 2 was dominated by figure‐8 conformations with self‐crossings. Cluster 3 contained more compact open structures, consistent with distorted circles or ovals, and Cluster 4 was enriched in elongated rod‐like molecules with less smooth, irregular backbones.

We then evaluated each cluster by measuring the proportion of molecules with at least one close MD counterpart (distance ≤ 0.03), allowing us to identify which groups of experimental conformations were well represented by the simulation and which were poorly covered. Cluster 1 contained 65 molecules, of which 92% were represented in the MD trajectory. Cluster 2 contained 30 molecules with 60% represented. In contrast, Cluster 3 (82 molecules, 44% represented) and Cluster 4 (54 molecules, 2% represented) were poorly represented within the MD simulation trajectory. These discrepancies suggest that while the MD model recapitulates some major conformational families observed by AFM, it does not yet fully capture the full conformational landscape observed through AFM, particularly the more compact and rod‐like shapes. This type of analysis enables us to directly assess the limited ability of simulations to sample the full range of representative experimental conformations and to identify potential imperfections in the simulation parameters, particularly when describing conformations not previously explored.

Representative splines from each cluster are shown, colored green if they have at least one close MD counterpart *PD* ≤ 0.03) and grey if they do not. Percentages denote the overall fraction of molecules in each cluster that were represented within the MD trajectory. Numbers above each spline indicate the minimum Procrustes distance to its closest MD counterpart.

## 4 Discussion

Here we have developed the first automated pipeline to describe and classify structures from AFM images by their conformation. By quantifying geometric similarity directly from molecular contours, this approach captures subtle conformational differences that are otherwise obscured when using conventional ensemble‐based methods such as gel electrophoresis, or classical feature‐based descriptors. Because this approach takes contours or shape boundaries as input, it generalizes naturally to more complex conformations where structural groupings are less apparent by eye, as well as to other sample types such as cells.

As the first application of Procrustes shape analysis to AFM images, we used our pipeline to map the conformational landscape of 339 bp DNA minicircles, as a system for which there is plentiful experimental and simulation data. Consistent with previous findings, our results demonstrate that DNA supercoiling promotes the formation of compact, self‐crossing conformations in DNA minicircles. Nonetheless, even among highly supercoiled species (such as Δ*Lk* = −4 and ‐6), a fraction of molecules retains more open conformations characteristic of relaxed topoisomers. This could in part be due to defects induced by supercoiling that relieve torsional stress and allow DNA to partially relax. In contrast, other supercoiled molecules become increasingly writhed and compacted when superhelical stress can no longer be dissipated through the formation of defects [16], resulting in a larger proportion of figure‐8 conformations for maximally supercoiled topoisomers.

Furthermore, visualization of molecular relationships through multidimensional scaling of the Procrustes distance matrix revealed that DNA minicircles form a continuous conformational spectrum, rather than discrete groupings. Data‐driven embedding captured gradual shape transitions without imposing predefined structural classes.

MD simulations showcase the conformational landscape through which a frustrated, closed loop DNA structure, e.g., 339 base‐pair minicircle can move, providing insight into the continuum of shapes each molecule can adopt. Conversely, as a surface technique, AFM captures a static snapshot of all the molecules that exist within an individual topoisomer population. By imaging hundreds of molecules, we captured the distribution of shapes that exist within a single topoisomer population, showing that they do not adopt a single, discrete shape. As the minicircles move through the conformations with minimum change in Procrustes distance we can infer dynamic structural changes as we are not seeing vast shape transitions. Minimal structural changes were measured in the relaxed population, Δ*Lk* = 0, implying that the dynamic structural changes observed in topoisomers with reduced linking, e.g., Δ*Lk* = −4 is induced by negative supercoiling, Whilst only a static snapshot of the minicircle conformations, the observed distribution reflects the underlying dynamics of the molecule and reflects a sampling of the free‐energy spectrum demonstrated by the MD simulations.

The strong correlation between Procrustes‐based and descriptor‐based geometric analyses suggests that the observed differences reflect genuine conformational changes. Both approaches revealed a progressive divergence from the relaxed state with increasing negative supercoiling, confirming that torsional stress systematically drives molecular compaction and self‐crossing. This agreement across fundamentally different analytical frameworks demonstrates that the Procrustes distances capture biologically meaningful variation in DNA conformation, with clusters identified in shape space corresponding to interpretable geometric descriptors such as radius, minimum Feret distance, and number of crossings. Because the Procrustes framework quantifies similarity across entire molecular contours rather than a limited set of predefined geometric features, it remains sensitive to even subtle heterogeneity that may be overlooked by feature‐based analyses.

Despite the accuracy of this method for describing and classifying shape, it requires that an object in an AFM image is faithfully segmented and splined before use, and also must compare every object for similarity before the model can be implemented. We therefore trained a neural network to perform this feature extraction, providing it with information on conformation to ensure that embeddings extracted from it focus more on molecular geometry than other image features. This model can be applied to unseen AFM data without the need for masking and splining. Not only is this faster, but could also be applied to a variety of topologically complex DNA substrates including: non‐canonical DNA helices such as Z‐DNA, G‐quadruplexes and cruciforms, longer closed‐loop DNA structures, e.g., bacterial plasmids and interlinked DNA structures such as knots, catenanes for which the molecular architecture is challenging to determine.

Having used these models to describe the continuous conformational spectrum of DNA minicircles from experimental AFM datasets, we could then compare these to simulated data of the same system, which provides molecular snapshots of a molecule moving through its free energy landscape. We used the Procrustes distance matrix to compare molecular snapshots between the two modalities, and highlight areas where they did not agree. We see that there are fewer AFM images with 2 or more crossings compared to the simulated data, which we attribute to the adsorption of these molecules on a charged surface where they are able to partially equilibrate. This method also provides a measure of how well represented each simulated snapshot is in the AFM conformational ensemble, which could be used to steer future simulations and understand the origins of these structural differences.

In summary we have developed the first automated method capable of describing a full conformational landscape for isolated snapshots of single DNA molecules. By developing a neural network guided by this method, we have increased the potential applications of this model to a wide range of AFM images containing isolated (bio) molecules.

## Author Contributions

Conceptualization: Laura Wiggins, Max C. Gamill, Agnes Noy, and Alice L. B. Pyne. Data Curation: Laura Wiggins and Tobias A. Firth. Formal analysis: Laura Wiggins. Funding acquisition: Laura Wiggins, Agnes Noy, and Alice L. B. Pyne. Investigation: Laura Wiggins, Tobias A. Firth, and Alice L. B. Pyne. Methodology: Laura Wiggins, Tobias A. Firth, Max C. Gamill, Agnes Noy, Jonathan M. Fogg, Lynn Zechiedrich, and Alice L. B. Pyne. Project administration: Laura Wiggins, Agnes Noy, and Alice L. B. Pyne. Resources: Agnes Noy, Jonathan M. Fogg, Lynn Zechiedrich, and Alice L. B. Pyne. Software: Laura Wiggins, Max C. Gamill, and Alice L. B. Pyne. Supervision: Alice L. B. Pyne. Validation: Laura Wiggins and Tobias A. Firth, Visualization: Laura Wiggins and Tobias A. Firth. Writing – original draft preparation: Laura Wiggins and Alice L. B. Pyne. Writing – review & editing: Laura Wiggins, Tobias A. Firth, Thomas E. Catley, Agnes Noy, Jonathan M. Fogg, Lynn Zechiedrich, and Alice L. B. Pyne.

## Conflicts of Interest

The authors declare no conflicts of interest.

## Supporting information




**Supporting File**: smll73768‐sup‐0001‐SuppMat.pdf.

## Data Availability

The data that support the findings of this study are available from the corresponding author upon reasonable request.
