# Supplementary material for: Mapping the Conformational Landscape of DNA Minicircles Through Atomic Force Microscopy and Shape Space Analysis
Source: Small. 2026 May 14;22(37):e14267. doi: 10.1002/smll.202514267 (PMC13325704; doi:10.1002/smll.202514267)
Supplement: Supplementary file 1 — Supporting File: smll73768‐sup‐0001‐SuppMat.pdf. [file SMLL-22-e14267-s001.pdf]

Supplementary material for "Mapping the conformational landscape of DNA minicircles through atomic force microscopy and shape space analysis"

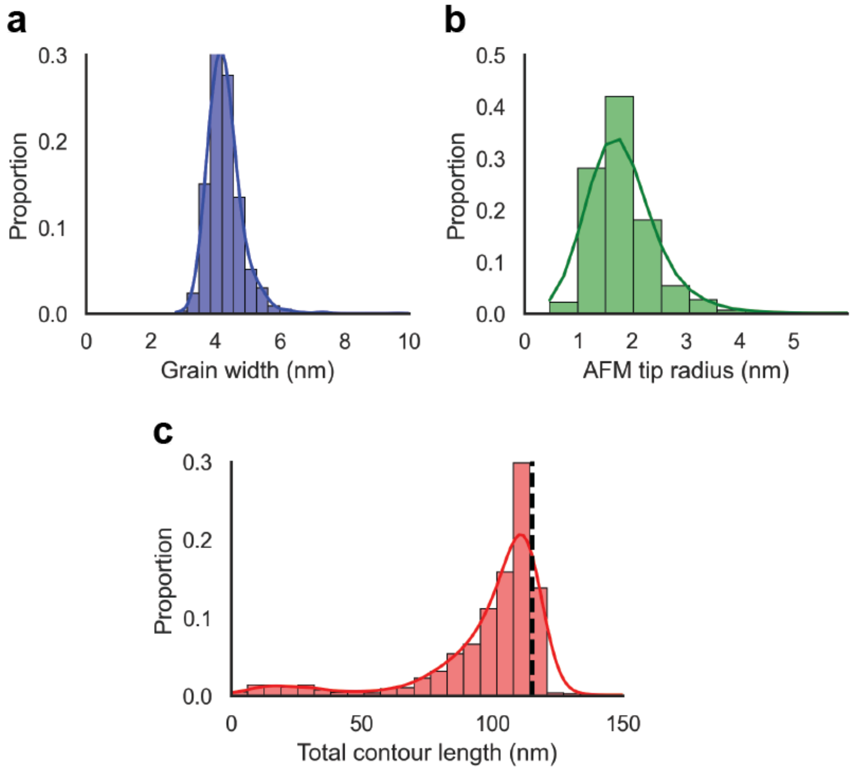

Supplementary Figure 1. Histograms/KDE plot of 339 base pair minicircle contour length and molecule grain width. a) Histogram of the mean grain width of the DNA, output from TopoStats processing, based on height thresholding. b) Distribution of tip radii calculated from the mean width of the grain mask and expected diameter of DNA (2 nm). c) Histogram of 339 base pair minicircle contour length which is calculated from the molecule spline.

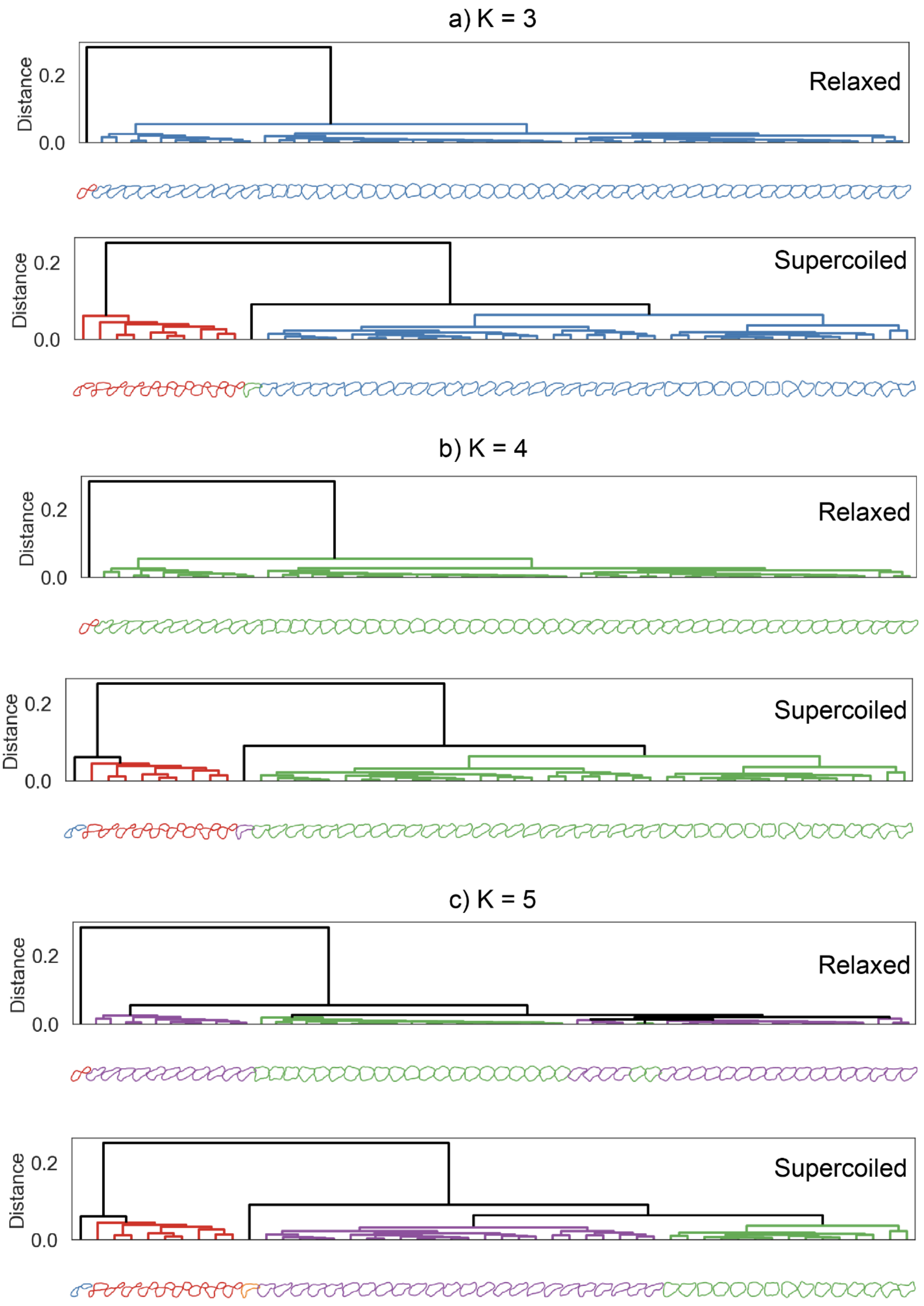

Supplementary Figure 2. Results of clustering with different values of K. One outlier spline is identified as its own cluster when K = 3 (shown in green), whereas another outlier spline is identified as its own cluster when K = 4 (shown in blue). When K = 5, the purple cluster appears to contain open shapes that are more compact than those in green.

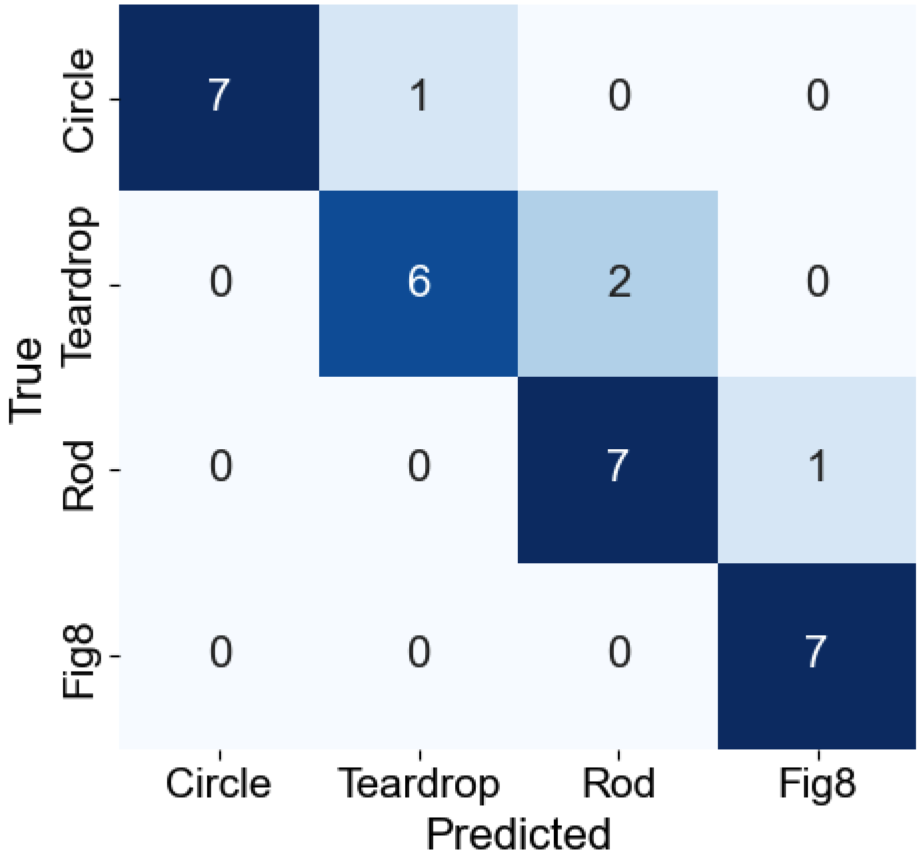

Supplementary Figure 3. A confusion matrix for the hold out test set when training an XGBoost classifier to identify circles, teardrops, rods and figures-of-8.

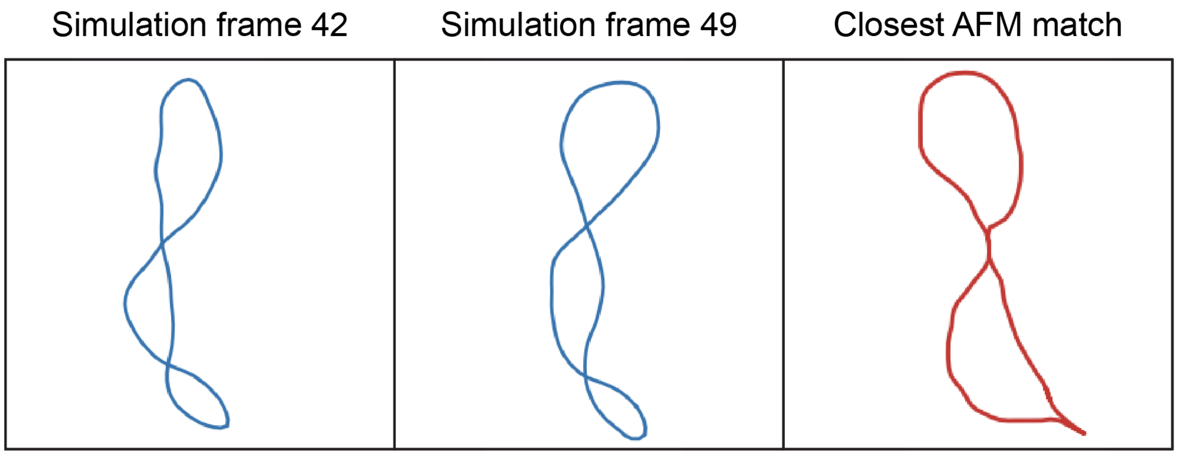

Supplementary Figure 4. Two simulation frames with 2 self-crossings and their closest AFM match, as determined by Procrustes distance. Note that the closest AFM match only has one crossing, thus does not share the same topology as the simulation frames, but their global geometries are similar.

Supplementary Table 1. List of geometric descriptors extracted through TopoStats and used within the feature-based analyses.

**Feature name**	**Description**
radius_min / radius_max / radius_mean / radius_median	The distance from the center to each pixel on the perimeter.
height_min / height_max / height_mean / height_median	The pixel values underlying the grain mask.
area	The area of the pixel-wise grain mask.
volume	Volume of the pixel-wise grain mask.
area_cartesian_bbox	The area of a box bounding the grain along cardinal directions.
smallest_bounding_width / smallest_bounding_length	The shortest bounding box length and perpendicular width of the grain in non-cardinal directions.
smallest_bounding_area	The area of the smallest possible box bounding the grain.
aspect_ratio	Ratio of the smallest bounding width to smallest bounding length.
max_feret / min_feret	The largest and shortest distance of the calipers rotating the grain between calipers (Feret diameters).
grain_endpoints	The number of pixels classified as endpoints (only 1 neighbour) in the pruned skeleton.

grain_junctions	The number of pixels classified as junctions (>2 neighbours) in the pruned skeleton.
total_branch_length	The sum of all branch lengths in the pruned skeleton.
total_contour_length	The total length of the detected contour or spline representing the grain boundary.
num_crossings	The number of crossover points detected along the skeleton.
grain_width_mean	The average width of the grain, measured across its central skeleton.
num_mols	The number of molecular entities (or grains).
average_end_to_end_distance	The mean linear distance between the endpoints of the skeleton.
min_crossing_confidence / avg_crossing_confidence	The minimum and average confidence values assigned to detected crossover points.

Supplementary Table 2. PERMANOVA results comparing topoisomer distributions pairwise. F statistics and q-values are reported following Benjamini–Hochberg FDR correction. Significant comparisons at FDR < 0.05 are indicated.

g1	g2	F	q-value	Significant (FDR 0.05)
-6	-3	18.50	0.0002	True

-6	-2	47.17	0.0002	True
-6	-1	85.10	0.0002	True
-6	0	176.33	0.0002	True
-4	-1	41.90	0.0002	True
-4	0	108.73	0.0002	True
-3	-1	24.51	0.0002	True
-3	0	77.47	0.0002	True
-2	0	37.17	0.0002	True
-4	-2	16.21	0.0008	True
-1	0	15.02	0.0018	True
-3	-2	6.84	0.0187	True
-6	-4	6.13	0.0276	True
-2	-1	5.38	0.0453	True

-4	-3	2.37	0.1482	False

Supplementary Table 3. Performance metrics derived from repeated stratified 5-fold cross-validation (20 repeats; 100 total model fits). Values are calculated from pooled out-of-fold predictions (n = 2,480).

Class	Precision	Recall	F1-score	Support
Circle	0.781	0.847	0.813	620
Fig8	0.930	0.972	0.950	600
Rod	0.818	0.863	0.840	640
Teardrop	0.767	0.626	0.689	620

Supplementary Table 4. Proportions of classes (Circle, Teardrop, Rod, and Fig8) identified within each topoisomer group (ΔLk = 0, –1, –2, –3, –4, –6). Values are reported as counts (n) and proportions relative to the total number of predictions per group.

**ΔLk**	**Class**	**n**	**Proportion**
0	Circle	104	0.395
0	Teardrop	98	0.373
0	Rod	55	0.209

0	Fig8	6	0.023
-1	Circle	52	0.230
-1	Teardrop	108	0.478
-1	Rod	52	0.230
-1	Fig8	14	0.062
-2	Circle	53	0.241
-2	Teardrop	88	0.400
-2	Rod	48	0.218
-2	Fig8	31	0.141
-3	Circle	44	0.199
-3	Teardrop	83	0.376
-3	Rod	51	0.231
-3	Fig8	43	0.195

-4	Circle	23	0.135
-4	Teardrop	56	0.329
-4	Rod	48	0.282
-4	Fig8	43	0.253
-6	Circle	33	0.143
-6	Teardrop	60	0.260
-6	Rod	61	0.264
-6	Fig8	77	0.333
